# A Nonoperative Approach for Neurosurgical Management of a Sylvian Fissure Dermoid Cyst

**DOI:** 10.7759/cureus.843

**Published:** 2016-10-24

**Authors:** Ali S Haider, Clarence Kee, Danielle L DeBacker, Ian T Watson, Eliel N Arrey, Tijani Osumah, Dean Leonard, Chen Chen, Maryam Alam, L. Gerard Toussaint III

**Affiliations:** 1 Texas A&M College of Medicine; 2 Health Science Center, Scott & White Clinic; 3 UT Houston Medical School, Memorial Hermann; 4 Ross University School of Medicine, Ross University; 5 General Surgery, Houston Methodist Hospital; 6 Neuroscience and Experimental Therapeutics, Texas A&M College of Medicine

**Keywords:** benign cystic teratoma, dermoid inclusion cyst, epidermoid cyst, intracranial cyst, neurosurgical management, sylvian fissure

## Abstract

The nonoperative management of sylvian fissure dermoid/epidermoid cysts presents a risk that is difficult to quantify. With rupture, potentially fatal complications such as chemical meningitis, hydrocephalus, fever, seizure, or meningeal irritation may occur. In this paper, we present an asymptomatic case of such a cyst with imaging evidence of prior rupture, and we review the literature for the likelihood of future complications. We use for illustration a case of a 68-year-old woman with imaging features of a sylvian fissure epithelial inclusion cyst who refused surgical intervention and review the literature for further investigation. Conservative management of our patient has not resulted in a complication in over five years, with the continued offer of surgical resection rejected by the patient. This article suggests the possibility of a safe, non-operative management of dermoid/epidermoid cysts of the sylvian fissure; however, the paucity of literature calls for larger studies yielding reliable data regarding the comparative risk of nonoperative management, including the rate of spontaneous rupture, versus the risk and complication incidence of surgical intervention.

## Introduction

Dermoid cysts are nonneoplastic, congenital ectodermal inclusion cysts, which include ectodermal derivatives such as apocrine, sweat and sebaceous cysts, as well as hair follicles and squamous epithelium. They are rare and benign tumors that account for less than one percent of all intracranial tumors [1]. Peak diagnosis of dermoid cyst tumors occurs in the third to fifth decade of life, either incidentally on a computed tomography (CT) and magnetic resonance imaging (MRI) for nonspecific symptoms or during the investigation of unexplained headaches, seizures or olfactory delusions. The presence and extent of symptoms depend on the location, size, and depth of the tumor as well as whether it is ruptured or intact. The symptoms of intact dermoid cysts include focal mass effect, hydrocephalus, and increased intracranial pressure. Ruptured dermoid cysts, however, can present with seizures, acute or chronic obstructive hydrocephalus, and rarely, chemical meningitis. This may lead to vasospasms, consequent transient ischemia leading to infarction and ultimately death [1]. While dermoid cyst tumors usually occur in the midline, they can seldomly also occur at the sylvian fissure. Unusual cases of that nature, therefore, warrant concern. Even though dermoid cysts are considered hypovascular tumors, the sylvian fissure is an area of rich vascularity [1]. Hence, an attempt at resection can lead to excessive hemorrhage. Categorically, radical resection of the tumor around critical neurovascular structures is generally not recommended [2]. To date, any studies investigating reported cases of sylvian fissure dermoid cysts and neurosurgical management have not yet been reported in the literature. Here, we present a unique case of a patient with a sylvian fissure dermoid cyst. Informed consent was obtained from the patient for this study.

## Case presentation

**Figure 1:**
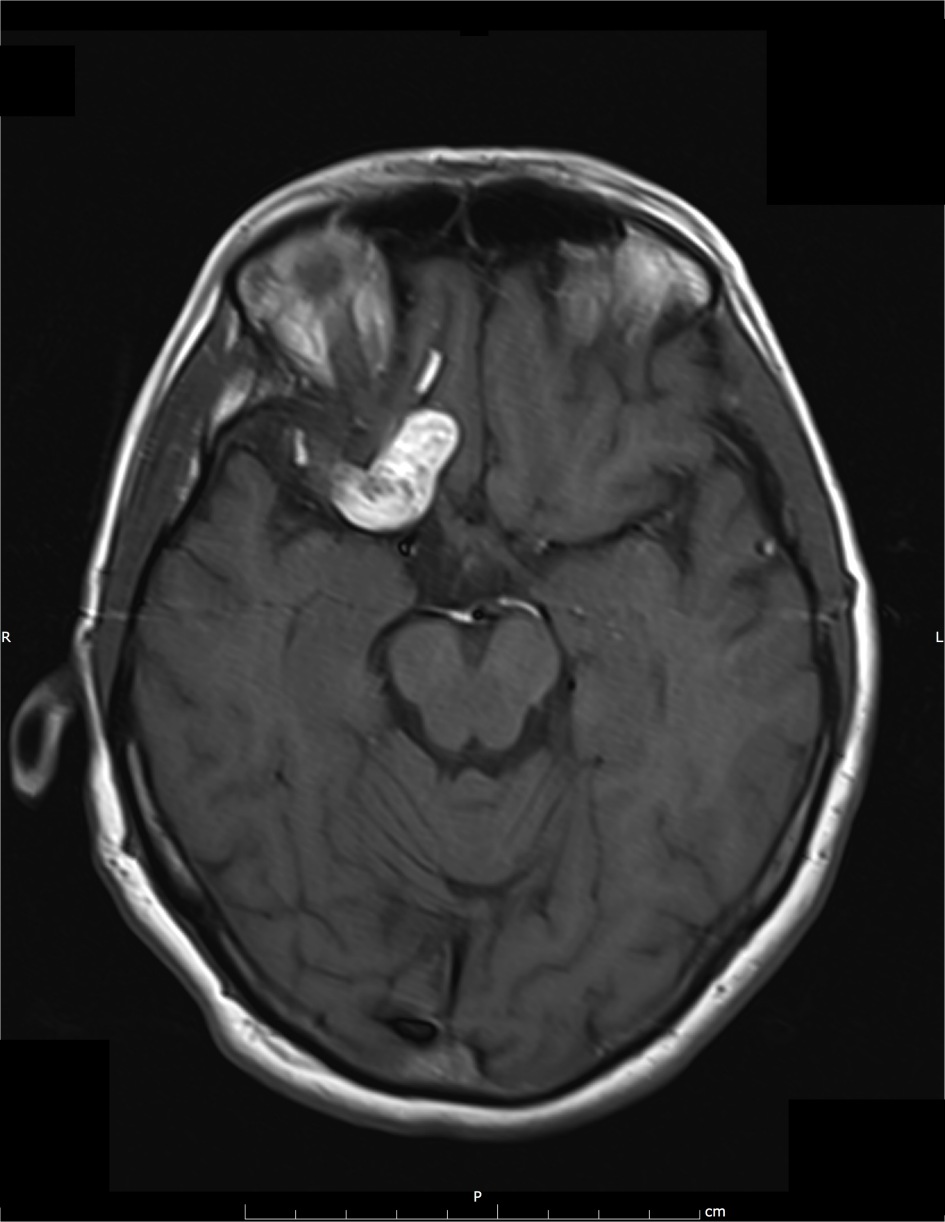
2008 Initial MRI This figure shows the MRI taken upon initial presentation in 2008. The image consists of a T1-weighted hyperintense mass in the right anterior cranial fossa. Also present are multiple small foci of T1-hypersensitivity within the right frontotemporal sulci suggesting prior rupture.

The patient is a 68-year-old right-hand dominant female referred for a brain lesion. She presented with complaints of intermittent fullness in her head primarily localized to the right temporoparietal area and accompanying dizzy spells. The patient elaborated by comparing her presenting symptoms to the sensation she gets when she goes in an airplane and her ears fail to equalize to the atmospheric pressure. The patient reported transient bouts of dizziness following bending and returning to an upright position. A previous MRI scan revealed a right middle and anterior fossa mass, which prompted differential diagnoses of a lipoma, a dermoid cyst or epidermoid inclusion cyst. She did not recall any traumatic brain injury, and she denied sudden sharp or shock-like sensations. A thorough neurological examination was negative. The patient was bright, alert, and articulate. Her memory was excellent. There were no deficits found in the cranial nerve function, motor or sensory systems, cerebellar function, cognition, or speech. The MRI of the brain revealed a T1-hyperintense boomerang-shaped mass (2.4 x 3.5 x 1.5 cubic centimeters) in the right anterior cranial fossa superior to the right orbital apex and superior to the lesser wing of the right sphenoid bone (Figure 1). A portion of the mass extends into the anterior portions of the right middle cranial fossa. In addition, the multiple small foci of T1-hypersensitivity were revealed within the right frontal temporal sulci, especially the right sylvian fissure, suggestive of an intracranial dermoid/epidermoid cyst that had previously ruptured. No invasion of cerebral or orbital tissue was noted, and there were no identifiable flares or T2 signal changes in the brain.

**Figure 2:**
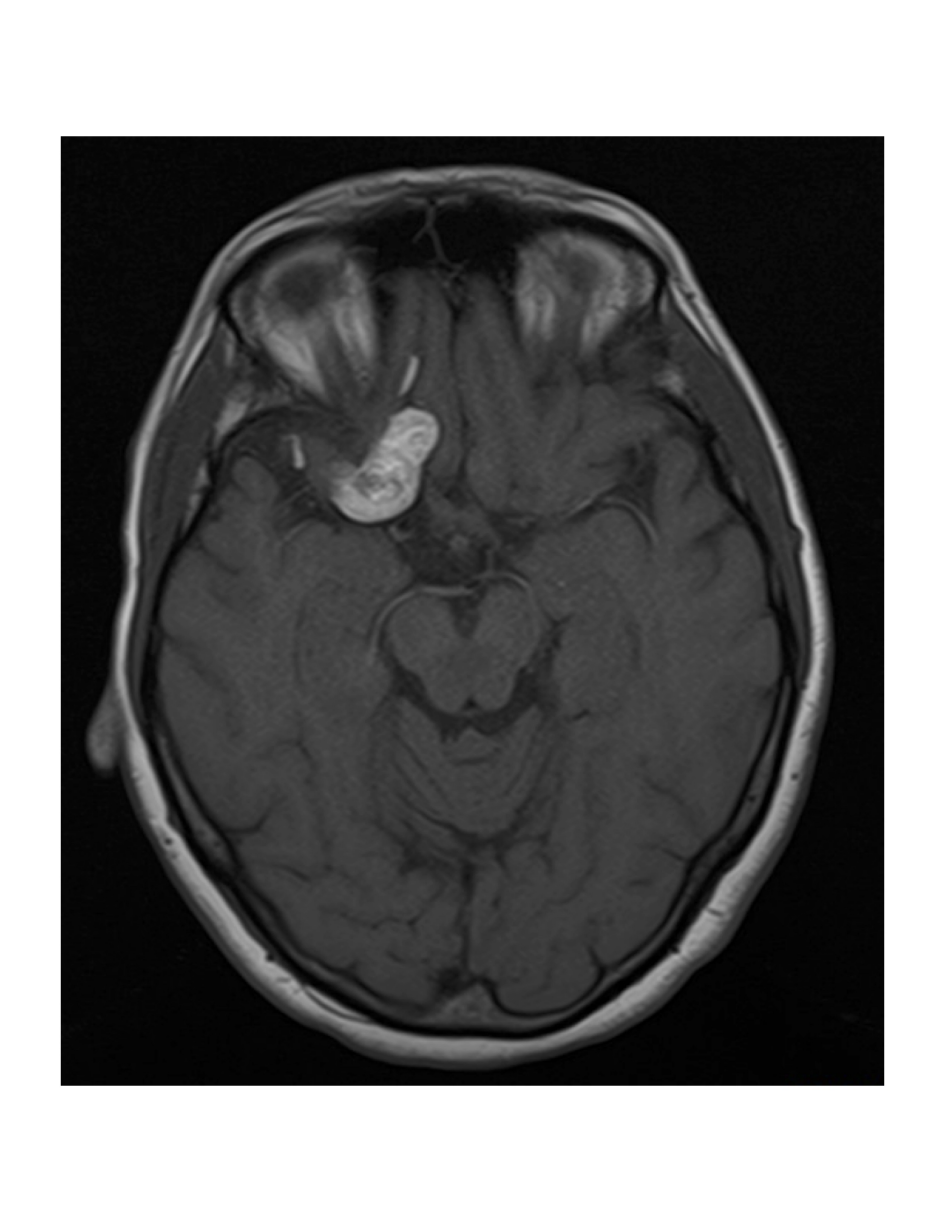
2009 Repeat MRI Repeat T1-weighted MRI image in 2009 showing no new evidence of significant change in appearance.

**Figure 3:**
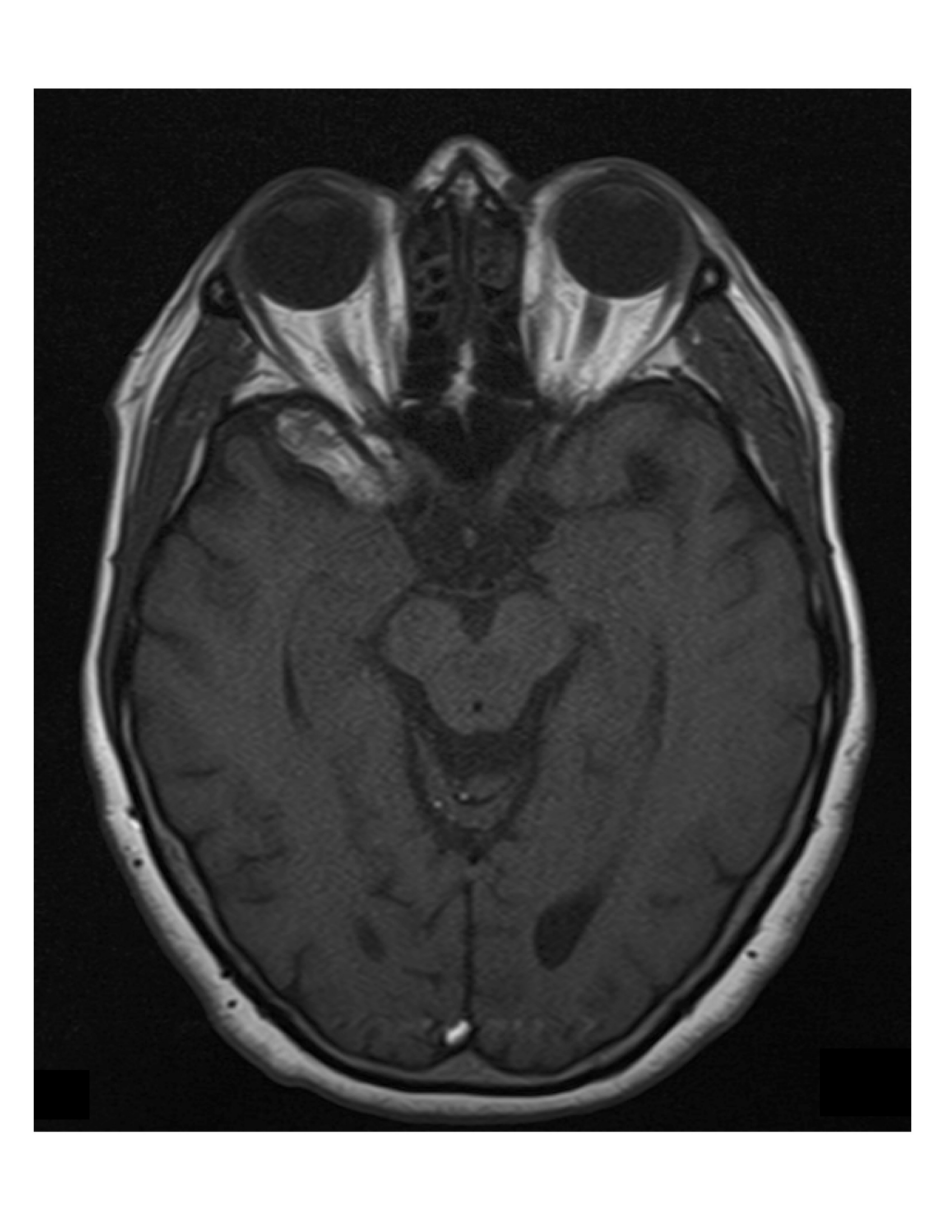
2014 Repeat MRI Repeat T1-weighted MRI image in 2014 showing no new evidence of significant change in appearance.

Rather, the mass appeared to be gently pushing on the brain. When these images were juxtaposed to the patient's previous MRI, it was observed that the dermoid/epidermoid cyst showed no evidence of significant structural or anatomical changes over time (Figures 2-3). However, a sole new finding was the gradient echo, a heme-sensitive axial sequence which demonstrated blooming and a drop in signal intensity throughout the main mass and within all T1-hyperintense foci located in the sulci. The signal drop suggests the presence of hemorrhagic products as it is not typically seen with oily substances often found within these cysts. No midline shift was noted; cerebral/lateral and third ventricles were consistent in size and shape with previous imaging.

More so, the intracranial CT showed a lesion comprised of fat attenuation in the right anterior middle cranial fossa. While this places a diagnosis of lipoma as a possibility, the presence of hemorrhagic regions around the lesion favored the characteristics of a dermoid/epidermoid cyst. A review of the previous CT images (chest and abdomen) were unremarkable, except for the presence of a left renal cyst.

## Discussion

There is a confusion about the differentiation of dermoids, epidermoids, and teratomas. The common misconception is that the epidermoids derive from the ectoderm, dermoids from the ectoderm and mesoderm, and teratomas encompass all the three germ layers. However, they are within the spectrum of congenital masses and are classified by origin and specific features. All the three have a squamous cell epithelium that provides a circumscribable barrier with a center of keratinaceous material [3-4]. The dermoid and epidermoid cysts are derived solely from the ectoderm and are true inclusion cysts [3-4]. The dermoids can be differentiated from the epidermoids by the involvement of complex tissues such as glands (sebaceous and sweat), hair follicles and other skin appendages (i.e. nails) that normally reside in the dermis [3-4]. There is a risk of malignant transformation, but this occurrence is exceptionally rare [5]. Unlike the epidermoids and dermoids, the teratomas are true neoplasms. The teratomas are foreign to the origin of growth and involve at least two layers of germ tissue. Nonetheless, up to 90% of the teratomas have all the three germ layers present [3]. The dermoid cysts comprise 0.3% of all brain tumors and are more commonly found near the midline [6]. The study found that 75% of the dermoid tumors are less than 10 mm, but can progress to substantial masses [7]. The most common locations are calvaria, intracranial, scalp, and within the spinal cord. Many dermoids are found in the pediatric population by palpation [8]. 

A study by Pryor, et al. in 2005 described that the majority of these dermoids were located in the periorbital region (61%) yet only four percent extended intracranially. Within the intracranial dermoids, the most common areas are suprasellar, sylvian fissure, cerebellopontine angle, basilar-posterior fossa, and within the ventricular system [6]. Our patient displayed a dermoid cyst in the sylvian fissure, which is exceedingly uncommon [4]. Patients with sylvian fissure dermoid cysts can also present with seizures, but our patient complained only of a feeling of fullness in her head [6]. The potentially fatal complications associated with a dermoid rupture are chemical meningitis, hydrocephalus, fever, seizure, or meningeal irritation [6]. Regardless, the risk of fatal complications is low. Moreover, there is evidence within the literature that epidermoid and dermoid tumors will rupture without a problem and spontaneously regress [9]. Though there are a number of studies highlighting surgical approaches to treating intracranial dermoid cysts at a variety of locations [10], there is a need for further studies to evaluate nonoperative approaches for patients who either refuse surgery or have dermoid cysts around critical structures in the brain. We continue to offer our patient surgical resection, which she refuses at each visit.

## Conclusions

The natural progression of epidermoid and dermoid cysts is not fully understood. Patients with a mass in a fundamental area of the brain are at a higher risk for surgical complications. Our findings suggest that nonsurgical management of sylvian fissure dermoid cysts is a viable clinical option; however, there is a paucity of information regarding this topic in the literature and larger studies are needed to further elucidate the risks and benefits.
